# Label-Free LC-MS^e^ in Tissue and Serum Reveals Protein Networks Underlying Differences between Benign and Malignant Serous Ovarian Tumors

**DOI:** 10.1371/journal.pone.0108046

**Published:** 2014-09-29

**Authors:** Wouter Wegdam, Carmen A. Argmann, Gertjan Kramer, Johannes P. Vissers, Marrije R. Buist, Gemma G. Kenter, Johannes M. F. G. Aerts, Danielle Meijer, Perry D. Moerland

**Affiliations:** 1 Department of Gynecology, Academic Medical Center, University of Amsterdam, Amsterdam, the Netherlands; 2 Department of Genetics and Genomic Sciences, Icahn Institute for Genomics and Multiscale Biology, Icahn School of Medicine at Mount Sinai, New York, New York, United States of America; 3 Clinical Proteomics Group, Department of Medical Biochemistry, Academic Medical Center, University of Amsterdam, Amsterdam, the Netherlands; 4 Waters Corporation MS Technologies Center, Manchester, United Kingdom; 5 Bioinformatics Laboratory, Department of Clinical Epidemiology, Biostatistics and Bioinformatics, Academic Medical Center, University of Amsterdam, Amsterdam, the Netherlands; Ospedale Pediatrico Bambino Gesu', Italy

## Abstract

**Purpose:**

To identify proteins and (molecular/biological) pathways associated with differences between benign and malignant epithelial ovarian tumors.

**Experimental Procedures:**

Serum of six patients with a serous adenocarcinoma of the ovary was collected before treatment, with a control group consisting of six matched patients with a serous cystadenoma. In addition to the serum, homogeneous regions of cells exhibiting uniform histology were isolated from benign and cancerous tissue by laser microdissection. We subsequently employed label-free liquid chromatography tandem mass spectrometry (LC-MS^e^) to identify proteins in these serum and tissues samples. Analyses of differential expression between samples were performed using Bioconductor packages and in-house scripts in the statistical software package R. Hierarchical clustering and pathway enrichment analyses were performed, as well as network enrichment and interactome analysis using MetaCore.

**Results:**

In total, we identified 20 and 71 proteins that were significantly differentially expressed between benign and malignant serum and tissue samples, respectively. The differentially expressed protein sets in serum and tissue largely differed with only 2 proteins in common. MetaCore network analysis, however inferred GCR-alpha and Sp1 as common transcriptional regulators. Interactome analysis highlighted 14-3-3 zeta/delta, 14-3-3 beta/alpha, Alpha-actinin 4, HSP60, and PCBP1 as critical proteins in the tumor proteome signature based on their relative overconnectivity. The data have been deposited to the ProteomeXchange with identifier PXD001084.

**Discussion:**

Our analysis identified proteins with both novel and previously known associations to ovarian cancer biology. Despite the small overlap between differentially expressed protein sets in serum and tissue, APOA1 and Serotransferrin were significantly lower expressed in both serum and cancer tissue samples, suggesting a tissue-derived effect in serum. Pathway and subsequent interactome analysis also highlighted common regulators in serum and tissue samples, suggesting a yet unknown role for PCBP1 in ovarian cancer pathophysiology.

## Introduction

Epithelial ovarian cancer is the leading cause of gynecologic cancer deaths in the Western world [1]. Approximately 70% of epithelial ovarian cancers are detected at an advanced stage. Although about 80% of the patients have complete remission of the disease after treatment with extensive debulking surgery and chemotherapy, the recurrence rate is very high. Currently, there are no curative treatment options for patients with recurrent disease and the 5-year survival rate is less than 30% [1]. In order to improve upon this poor survival rate many studies have tried to identify more sensitive early detection markers and methods for discriminating between different pelvic masses [2], [3]. A large number of these studies used various mass-spectrometric methods to search for new markers in patient material such as serum [4]. The OVA1 test, which has been approved by the Food and Drug Administration (FDA) in 2009, was one of the first multimarker diagnostic tests that resulted from this type of research [5]. However, when Moore et al. [6] evaluated several of these biomarkers alone and in combination with CA 125 in prediagnostically collected sera from women in the Prostate, Lung, Colorectal and Ovarian Cancer Screening trial the addition of these biomarkers to CA125 did not improve sensitivity for preclinical diagnosis. Other strategies involve a combination of known serum biomarkers such as Human Epididymal secretory protein 4 (HE4) and CA125 in a discriminatory algorithm such as ROMA (Risk of Ovarian Malignancy Algorithm) [7]. Most of the biomarkers being investigated or used in the clinic today are serum-based proteins, which are logical targets both for use as biomarkers for screening and diagnosis, as well as potential drug targets. However, most of the recently found biomarkers in serum or plasma are acute phase proteins that are not specific for one type of cancer or disease [8]. In fact, only a few FDA approved cancer markers in current clinical use are tumor-derived proteins (e.g., prostate-specific antigen, carcinoembryonic antigen), and are present in serum at very low concentrations (1-10 ng/mL) only [9]. In this study we have tried to overcome the problematic aspecific aspects of blood-based protein markers by aiming to identify proteins differentially expressed between tumor tissue samples of patients with a serous adenocarcinoma of the ovaries versus a benign serous tumor. In tumor tissue the potential marker proteins are present at much higher concentrations, which could facilitate protein identification. By comparing the protein content of tumor tissue with that of serum samples, we aimed to detect or infer reliable tumor-produced serum biomarkers. Directly studying tumor tissue, even though it enhances the probability of finding tumor-derived markers, is challenging nevertheless. Accurate analysis of tumors is often hampered by within-tumor heterogeneity, for example due to the presence of contaminating stroma cells, necrosis or infiltrating lymphocytes [10]. Laser microdissection minimizes this problem via the rapid and reliable isolation of a specific cell population or type from a tissue section under direct microscopic visualization [11], [12].

Using laser microdissection we obtained homogenous tumor samples that were subsequently measured with a mass spectrometric approach called LC-MS^e^. LC-MS^e^ differs from traditional data-dependent acquisition (DDA) modes in that all precursor and fragment ions are measured by alternating the collision energy between a low (precursor ions) and elevated (fragment ions) profile without selection of ions. Thus LC-MS^e^ is able to identify and quantify more peptides in complex samples using a single dimension reversed phase ultra-high pressure liquid chromatography (UPLC) separation, than DDA on quadrupole time-of-flight instruments (QTOF) [13], [14].

The primary objective of the present study was to identify differentially expressed proteins in tissue and serum, comparing benign and malignant serous ovarian tumors. After initial protein identification we performed extensive pathway and network analyses in order to find differences in the underlying protein pathways associated with benign and malignant ovarian tumors.

## Experimental Procedures

### Patients and Ethics Statement

After written informed consent was obtained, serum and tissue samples were prospectively collected from patients admitted at the Academic Medical Center (AMC) for treatment of an ovarian tumor. The study was performed in agreement with the Helsinki Declaration and approved by the Ethical Committee at the Academic Medical Center, University of Amsterdam. For serum analysis, we included 6 patients that were newly diagnosed with non-familial invasive serous epithelial ovarian carcinoma, stage IIIB or higher based on FIGO (Fédération Internationale de Gynécologie Obstétrique) criteria, and 6 patients with benign serous cystadenomas. For 4 of the 6 patients with benign disease and 3 of the 6 with a malignant tumor, also tissue was available. To increase sample size for the tissue comparison, we included two additional patients with benign disease and two additional patients with a malignant tumor. Both in serum and tissue, the two groups were matched for age, body mass index (BMI), menopausal status and sample-storage duration. Clinicopathological data are listed in Table 1. All samples were collected using a strict protocol. Blood was collected from all patients by the same operator, at least two hours after the patient's last meal, and left to clot for 30 minutes. After centrifugation (at 1750×g) serum was immediately frozen and stored at -80°C. Samples used for these experiments were only thawed once.

**Table 1:** Patients characteristics.

	Serum		Tissue	
	*Malignant*	*Benign*	*Malignant*	*Benign*
Patients	6	6	5	6
Age (mean, SD)	53 (16.5)	57 (7.2)	51 (15.2)	56 (9.8)
BMI (mean, SD)	25 (4.9)	25 (5.1)	23 (3.3)	26 (4.8)
Pre-menopausal	3	1	2	2
Post-menopausal	3	5	3	4
CA125 kU/L (median, range)	6946 (113–14100)	12 (7–26)	2651 (113–7737)	70 (7–376)
Differentiation grade				
2	1		1	
3	5		4	
**Figo Stage**				
III	5		5	
IV	1			

Clinicopathological characteristics of the patient groups. SD: standard deviation.

### Laser microdissection and protein isolation

During surgery tumor tissue was collected, snap frozen in liquid nitrogen, and stored at −80°C within 30 minutes of surgery. From these samples 10 µm cryostat sections were prepared. One section was stained with hematoxylin and examined microscopically in order to detect tissue areas of interest for microdissection. Corresponding consecutive tissue sections were mounted on a microscope slide coated with a membrane (polyethylene naphtalate (PEN) Zeiss/Palm, Bernried, Germany) and stored at −80°C. Comparison of stained and unstained tissue sections revealed that hematoxylin staining had no influence on the quantitative protein measurements and identification using LC-MS^e^.

**Figure 1:**
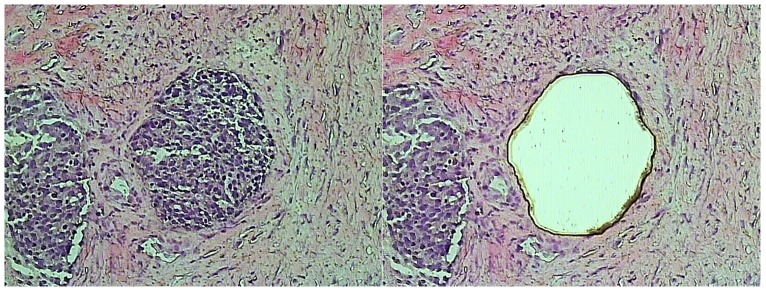
Laser capture microdissection of tumor tissue. A 10 µm hematoxylin stained cryostat section of a serous adenocarcinoma of the ovaries. The picture on the left is prior to microdissection, on right the same slide after microdissection is shown.

Tissue areas were cut using a Veritas Microdissection System (Arcturus Molecular Devices, CA, USA), as described earlier [15]. Slides were stained for 1 minute with hematoxylin. Dissected cancer samples contained at least 90% cancer cells (Figure 1). Using microdissection, the samples from benign cystadenomas were enriched for 75%–90% epithelial cells.

We obtained 100,000 cells from each sample. Cells were denatured in 20 µl 0,1% RapiGest detergent solution (Waters Corp., Milford, MA) and heated at 80°C for 15 minutes. After centrifugation at 1750×g for 10 min, the supernatants were collected. Several tests were performed to determine optimal lysis conditions. Protein concentrations were measured using a Bicinchoninic Acid (BCA) solution (Sigma-Aldrich Chemie GmbH, Schnelldorf, Germany) and 4% CuSO4. The average protein concentration obtained from 100.000 cells was 1 mg/ml.

### Sample preparation for LC-MS analysis

Serum samples were diluted in 50 mM ammoniumhydrogencarbonate (Fluka), 1% RapiGest SF (Waters Corp., Milford, MA) to ∼10 µg/µl total protein concentration. Laser dissected tissue samples were lysed in 50 mM ammoniumhydrogencarbonate, 0.1% RapiGest SF, prior to protein determination by BCA-assay (Thermo-Scientific) according to the manufacturer's protocol. Subsequently serum samples were denatured at 80°C for 15 min and tissue samples at 95°C for 10 min prior to reduction of disulfide bridges 5 mM dithiorthreitol at 60°C for 30 min. Free sulfhydryl groups were alkylated by addition of 15 mM iodoaceteamide and incubation at ambient temperature for 30 min. in the dark. Samples were digested overnight by addition of 0.3 activity units/µg total protein content of sequencing grade modified trypsin (Promega, Madison WI) and incubation overnight at 37°C. Following digestion the acid-labile detergent was hydrolyzed by incubation in the presence of 0.5% trifluoroaceticacid (Biosolve, the Netherlands) at 37°C for 45 min and removal of immiscible debris by centrifugation at 20.000×g for 10 min, collecting the supernatant. Prior to analyses, serum digest samples were diluted with aqueous 0.1% formic acid solution (Biosolve, the Netherlands) to ∼0.5 µg/µl total protein concentration. Subsequently both serum digest and tissue digest samples were mixed 1∶1 with a Mass Prep Quantitation standard (Waters, Milford MA) for quantitation purposes (mix with either 100 fmol/µl Enolase or Alcohol dehydrogenase 1 from *S. cerevisiae* for tissue samples or 250 fmol/µl ClpB from *Escherichia coli* for serum samples).

### LC-MS analysis

0.5 µg of total protein was loaded onto a Nano-Acquity system (Waters Corporation) equipped with a Bridged Ethyl Hybrid C18 1.7 µm, 15-cm×150-µm analytical reversed phase column (Waters Corporation) and operated at a column flow rate of 1 µl/min. All samples were measured in triplicate. Apart from the column dimension and flow rate, all other gradient conditions were as detailed earlier [14]. Analysis of tryptic peptides was performed using a Synapt G2 quadrupole time of flight mass spectrometer (Waters Corporation, Manchester, UK) with the operating and experimental conditions as previously described [14]. Accurate mass precursor and fragment ion LC-MS data were collected in data-independent MS^e^ mode of acquisition. This method alternates the energy applied to the collision cell of the mass spectrometer between a low and elevated energy state [16], [17]. Briefly, the low energy portion of the obtained data sets is typically used for quantification of the proteins, whereas the combined low and elevated energy information are utilized for identification purposes. In both modes of acquisition, mass spectral information was obtained from *m/z* 50 to 1990 at a resolving power of at least 10,000 full width half maximum.

### Data processing and protein identification

Continuum LC-MS^e^ data were processed and searched using ProteinLynx Globalserver version 2.5 (PLGS 2.5, Waters Corporation). Protein identifications were obtained with the embedded ion accounting algorithm [18] of the software and searching the human SwissProt entries of the UniProt database (release 13.2) that was modified to include N-terminal processing of proteins using the protein maturation device software [19] and to which enolase and alcohol dehydrogenase 1 of *S. cerevisiae* or ClpB from *Escherichia coli* were appended as the internal standard to provide the ability to address technical variation and to accommodate concentration determinations [13]. The search tolerances were set to automatic, typically 10 ppm for precursor and 25 ppm for product ions, cysteine carbamidomethylation specified as a fixed modification and N-terminal acetylation, deamidation of asparagine and glutamine and oxidation of methionine as variable modifications. Estimation of false positive identification rates was performed by searches in a shuffled version of the UniProt human protein database generated in PGLS 2.5. Robust criteria were applied for quantification, including the identification of minimally three and seven product ion matches per peptide and protein, respectively. In addition, at least two peptides per protein had to be identified and the identification had to occur in at least two independent patient serum or tissue samples. Protein false positive identification rate, taking into account the criteria mentioned above, was less than one percent. Label free quantitation of proteins was based on the sum of the signal intensities of the three most abundant peptides of a protein, divided by the sum of the signal intensities of the three most abundant peptides of the internal standard, times the amount in fmol of standard injected on the column. This gives an estimation of the molar amount of each protein injected on the column. PLGS 2.5 determines the molar amount (the amount in ng is determined using the molecular weight in the database) for each protein based on the ratio of its three most abundant peptides (HI3) determined in each individual experiment [13]. These measured amounts were used for proteins that met the criteria for identification indicated above in order to calculate the average concentration of each protein in g/L using the dilution factor of the samples. Protein identity and quantitative data were exported as a comma separated value file for further statistical and pathway analysis. The mass spectrometry proteomics data have been deposited to the ProteomeXchange consortium (http://proteomecentral.proteomexchange.org) [20] via the PRIDE partner repository with the dataset identifier PXD001084. A list of filenames deposited in ProteomeXchange and their corresponding sample annotation can be found in the supporting information section (Table S1, worksheet ‘Filenames’).

### Statistical analyses

Statistical analyses were performed using Bioconductor packages and in-house scripts in the statistical software package R [21]. Serum and tissue data were analyzed separately. Missing values in the raw quantitative data were imputed with the minimum value measured for the sample in which the missing value occurred. Data were scale normalized to the same mean intensity across samples; resulting values were then log2-transformed with an offset of 1 in order to stabilize their variance. The array Quality Metrics R package was used to assess whether all MS samples were of good quality. The tissue samples were measured in two separate batches; quality control clearly showed the presence of a pronounced batch effect in the normalized data. Tissue data were made comparable across batches by fitting a linear model, including both batches and regular conditions, and removing the component due to the batch effects (function ‘removeBatchEffect’ from the R package limma). After normalization and batch correction (tissue study), the technical replicate samples for each patient were highly similar. A more detailed description of the quality control analysis is given in Text S1. The technical replicates of the batch corrected data were averaged and resulting data was used for hierarchical clustering analyses. For benign tissue sample 4 only two technical replicates were used for data analyses due to technical problems with the third measurement.

For each identified protein a linear model was fit on the normalized data containing the two conditions (benign and malignant) as explanatory variable; for the tissue study a batch factor was also included in the linear model. A consensus between-replicate correlation was estimated for the technical replicates (function ‘duplicateCorrelation’ from the R package limma) and included in the linear model fit. Differentially expressed proteins between benign and malignant tumors were detected using a moderated t-test. P-values were adjusted for multiple-testing using the Benjamini-Hochberg false discovery rate [22]. Proteins were considered to be significantly differentially expressed between the two conditions with an adjusted p-value<0.05, and a fold change ≤−1.4 or ≥1.4. Moreover, the protein had to be present before imputation in at least 50% of the samples in at least one of the two conditions.

### Hierarchical clustering and enrichment analyses

Two-dimensional clustering (Pearson correlation, average linkage) was performed on protein expression values using the function ‘heatmap.2’ from the R package gplots [23]. The Cytoscape plugin “ClueGO” v2.0.2 was used for protein set enrichment analysis by uploading the list of proteins with their UniProt IDs and using a custom background reference set consisting of all proteins detected in the experiment. [24], [25]. For exploratory pathway enrichment analyses a less stringent cutoff, using an unadjusted p-value less than 0.05 was used. Lists of serum or tumor proteins differentially expressed between malignant and benign cases were submitted for protein set enrichment analysis according to the gene ontology (GO) biological process domain. Within ClueGO we used the ‘compare lists’ feature and compared protein set enrichment (one-sided hypergeometric test) for the differentially expressed serum and tumor proteomes simultaneously. The edges of the resulting ClueGO network are based on kappa statistics and reflect the relationships between the GO terms (network nodes) based on the similarity of their associated proteins.

Protein accession numbers and their corresponding fold changes were imported into the web-based integrative software MetaCore (v 6.8 build 30387; Thomson Reuters, St. Joseph, MI) for network analysis [26]. MetaCore analysis was used for network enrichment and interactome analysis using the differentially expressed proteins (unadjusted p-value<0.05) in either the serum or tissue samples of benign versus malignant samples with the background reference set consisting of all proteins detected in the experiment. Relative connectivity of proteins inside a set (intra-connectivity) and between a set and the global interactome (inter-connectivity) were calculated using MetaCore protein interaction database. A ranking of importance was given by using a “knowledge-based” analysis that considers the differentially expressed proteins in the context of their known interactors in complex protein and molecular networks.

## Results

### Hierarchical clustering of serum and tissue proteome

**Figure 2:**
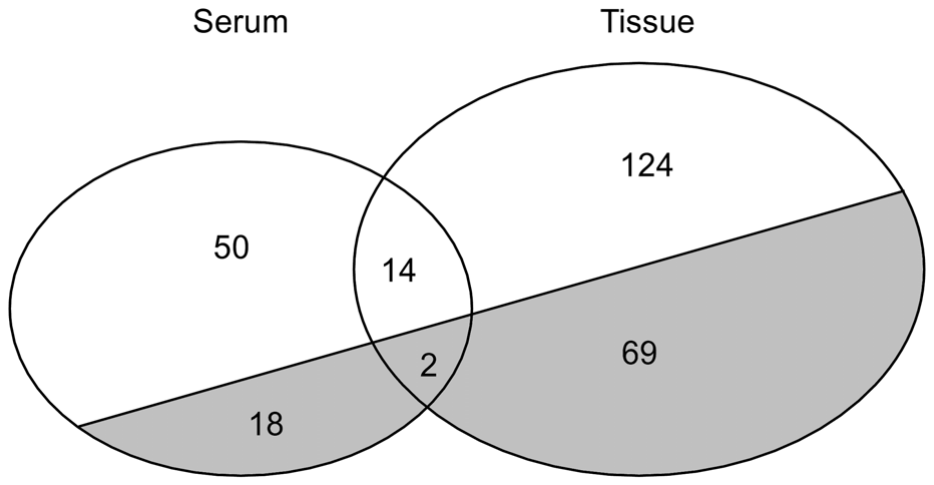
Venn diagram of the detected proteins. We detected a total of 84 proteins in serum and 209 in tissue, present in at least 50% of the samples in at least one of the conditions (benign or malignant). The grey area represents the proteins with an adjusted p-value of <0.05 when comparing benign with malignant.

In total, we identified 84 proteins in serum and 209 in tissue, which were present in at least 50% of the samples in at least one of the conditions (benign or malignant) (Table S1). Of those proteins 20 and 71 were significantly differentially expressed between benign and malignant disease in serum and tissue samples, respectively (Figure 2). Only 16 proteins were detected in both serum and tissue samples (highlighted in yellow in Table S1). Of these, only two proteins had an adjusted p-value of <0.05, Apolipoprotein A-I and Serotransferrin.

**Figure 3:**
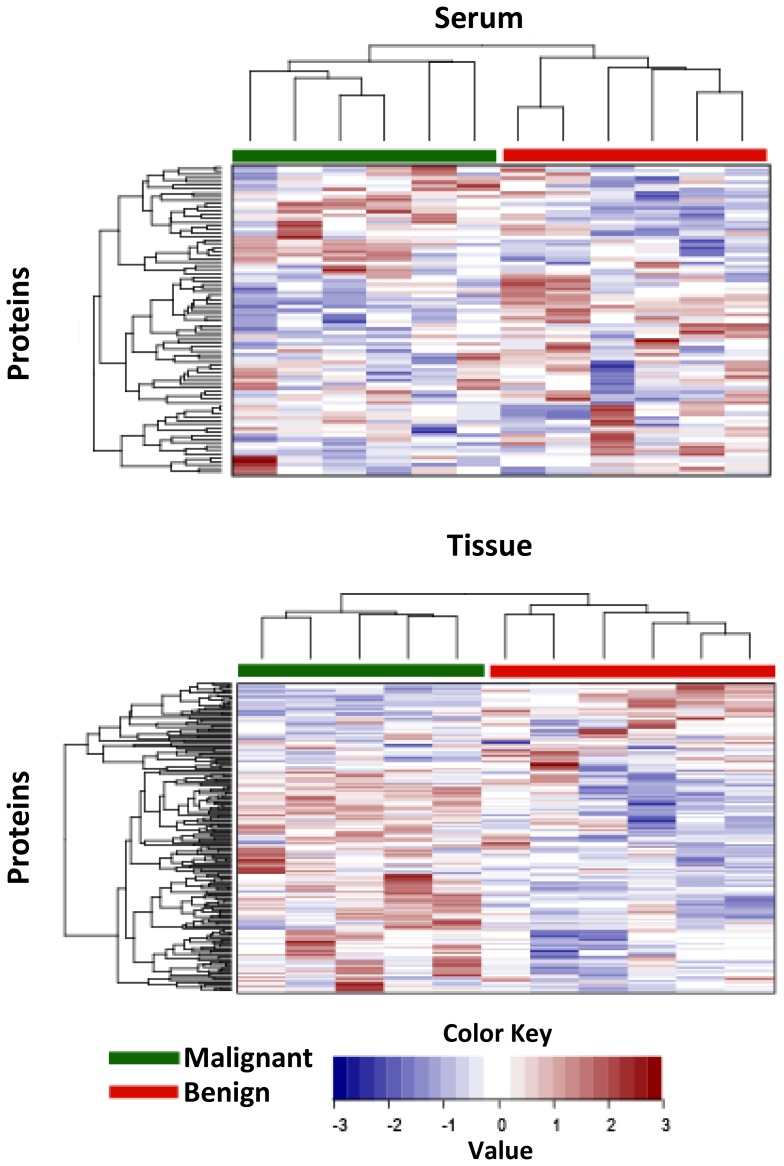
Unsupervised hierarchical clustering. Unsupervised hierarchical clustering was performed of the 84 and 209 proteins detected in serum and tumor tissue samples, respectively. The key color bar indicates standardized protein expression levels (dark red indicates relatively higher expression; dark blue indicates relatively lower expression).

Hierarchical cluster analysis was used to group serum and tumor samples according to the similarity of their expression profiles. Clustering the 84 and 209 proteins detected in serum and tumor samples respectively, could perfectly separate the benign and the malignant tumors (Figure 3). This illustrates that malignancy was the strongest signal in this dataset and that the benign and malignant samples constitute well-defined groups that could reveal differences in underlying biology between the processes leading to malignancy. This result also supported our methodology in which we minimized heterogeneity by going through detailed patient and sample selection and by microdissecting the tumor tissue.

### Biological functions and networks enriched in serum and tumor signatures between malignant and benign samples

**Figure 4:**
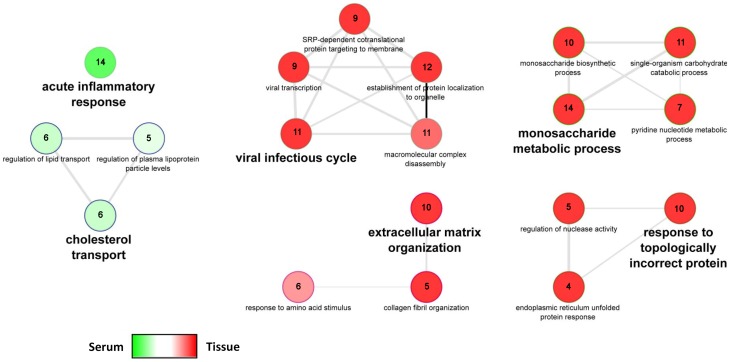
Protein set enrichment analysis using the Cytoscape plugin ’ClueGO’. In the network, only significantly enriched categories (p-value<0.05, Bonferroni corrected) are shown. The node color represents the commonality of members of either the differentially expressed serum or tumor proteome list. Dark red highlights categories that are specific to the tissue proteome signature while dark green represents biological processes that are specific to the serum proteome signature. The number of proteins associated with each GO category are indicated within the corresponding node. The edges of the resulting ClueGO network are based on kappa statistics and reflect the relationships between the GO terms (network nodes) based on the similarity of their associated proteins. The complete results and relevant statistics are summarized in Table S2.

To explore and compare the biological processes that contribute to changes of the serum and tumor proteome during ovarian cancer development, a protein set biological enrichment analysis was performed on the differentially expressed protein signatures derived from the serum and tissue comparisons. The serum proteome signature was significantly enriched in categories associated with immune function and lipoprotein metabolism. In contrast, the tumor proteome signature was significantly enriched in categories associated with glucose metabolism and the unfolded protein response (Figure 4; Bonferroni corrected p-value<0.05). Interestingly, there is little overlap in the biological processes enriched in serum and tissue signatures. The proteins associated with these protein sets and the relevant statistics are summarized in Table S2.

### Key transcriptional regulatory networks in ovarian cancer

**Figure 5:**
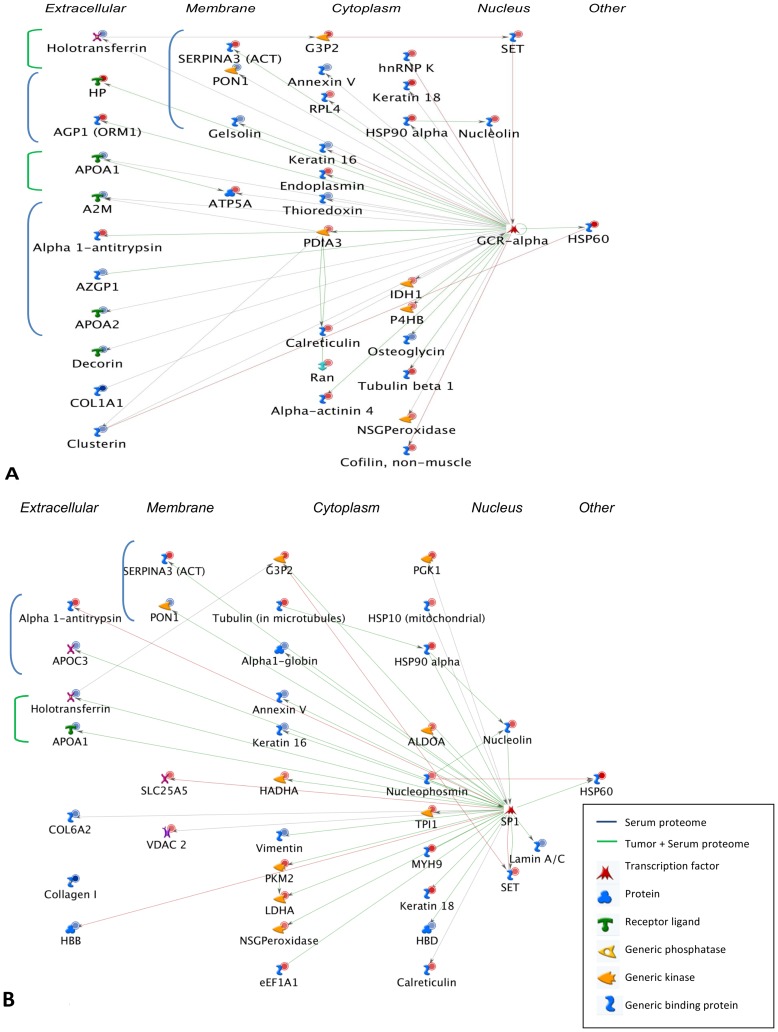
Proteins in the serum and tumor datasets that are potentially associated with GCR-alpha (A) and SP1 (B) pathways. The proteins marked in blue were found in the serum dataset, those marked in green in both tumor and serum. Unmarked proteins are specific for the tumor signature (except GCR-alpha and SP1). Proteins are ordered according to their position within the cell (extracellular, membrane bound, cytoplasmic or nucleic). Individual proteins are represented as nodes, the different shapes of the nodes represent the functional class of the proteins. The arrowheads indicate the direction of the interaction, the color of the lines between nodes describes activation (green), inhibition (red), and unspecified (black) interactions. The small circles on top of the protein symbols indicate up-regulation (red) or down-regulation (blue).

We subjected the lists of differentially expressed serum and tissue proteins to the transcriptional regulation network algorithm in MetaCore. This was used to infer potentially important transcription factors in the carcinogenic process. Summaries of the top scoring transcriptional regulatory networks and the key associated transcription factors in respectively serum and tissue are shown in Table S3. The most significant transcriptional regulatory network in serum was GCR-alpha (glucocorticoid receptor alpha) signaling. Eleven proteins that are upstream or downstream of GCR-alpha signaling were found differentially expressed in the serum of patients with a serous adenocarcinoma of the ovaries compared to patients with a serous cystadenoma. The pathways linked to GCR-alpha signaling may explain regulation of the acute inflammatory response that was seen altered in the cancer patients. In the tumor tissue, GCR-alpha was also highlighted in the top scoring transcriptional networks although associated with pathways related to cellular localization and protein folding (Table S3). Figure 5A summarizes, which proteins in our dataset are potentially regulated by GCR-alpha. Interestingly, despite the limited overlap in terms of differentially expressed proteins (Figure 2) and significantly enriched biological processes (Figure 4), two GCR-alpha target proteins, ApoA-1 and Serotransferrin, were present in the signatures of both the serum and the tumor proteome (Figure 5A). These results suggest that the serum cancer proteome could be used to detect changes in GCR-alpha tumor associated processes.

Sp1 was associated with the third top scoring transcriptional network in tumor tissue (Table S3) after the well-known cancer-related transcription factor c-Myc and also CREB-1 that was recently found to be associated with OVCA cell line platinum sensitivity and overall survival [27]. Sp1 was also associated with the top scoring transcriptional networks in the cancerous serum signature. In total, 32 proteins associated with the Sp1 transcriptional pathway were differentially expressed between benign and malignant tissue samples (Figure 5B). Biological processes associated with Sp1 included several pathways related to lipid metabolism such as regulation of cholesterol and sterol transport, and acute-phase response and response to endogenous stimulus. This suggests that this transcriptional regulator might be an important contributor to the metabolic reprogramming that occurs within the tumor as highlighted by our ontology analysis (Figure 4).

Overlap between candidate key-regulatory transcription factors in serum and tumor tissue as highlighted by the transcriptional regulatory network analysis was far greater than at the individual protein level. Nine of the top-20 significantly associated transcription factors in serum were also found in the top-20 of tumor tissue, among them well known cancer-associated transcription factors such as p53 and c-Jun.

### Key proteins differentially expressed between benign and malignant ovarian tumors

In an attempt to move beyond transcriptional regulation and find other regulatory proteins that are statistically significantly "overconnected" within our proteomics signatures, we subjected the list of differentially expressed proteins in ovarian tumor samples to “interactome analysis by protein function”. This helps in prioritizing potentially relevant individual proteins or hubs. Table 2 summarizes the results of the interactome analysis. Only five proteins were found to be significantly over-connected in the tumor proteome signature namely, PCBP1, 14-3-3 zeta, 14-3-3 beta, alpha actinin-4, and HSP60 (CH60/HSPD-1). These proteins were significantly upregulated in malignant tumors and have all been linked to cancer previously and all but one, PCBP1, specifically to ovarian cancer.

**Table 2:** Interactions by protein function.

Protein	Actual	n	R	N	Expected	Ratio	p-value	z-score
PCBP-1	11	108	13	296	4.743	2.319	0.000402	3.68
Alpha-actinin 4	14	108	19	296	6.932	2.019	0.000754	3.476
HSP60	17	108	27	296	9.851	1.726	0.003122	2.993
14-3-3 beta/alpha	34	108	56	296	20.43	1.664	3.71E-05	4.176
14-3-3 zeta/delta	55	108	97	296	35.39	1.554	5.33E-07	5.035

Interactions by protein function based on the connectivity with the tumor tissue signatures and with proteins from the human proteome MetaCore database. The proteins were considered over-connected when the number of observed interactions exceeded the number of expected interactions. Actual: number of network objects in the signature which interact with the chosen object; n: number of network objects in the signature; R: number of network objects in the background list which interact with the chosen object; N: total number of protein-based objects in the background list; Expected: mean of hypergeometric distribution. Ratio: connectivity ratio (Actual/Expected); z-score: (Actual-Expected)/(standard deviation); p-value: probability to have the value of Actual or higher (lower for negative z-score) by chance under null hypothesis of no over- or under-connectivity.

**Figure 6:**
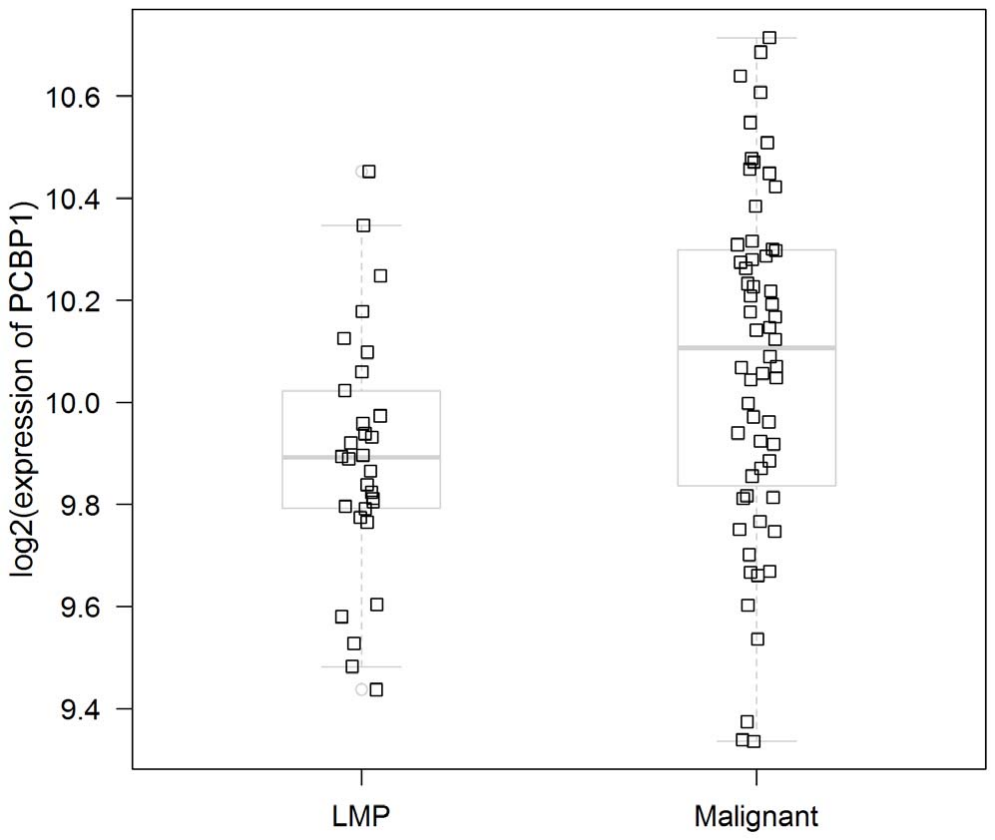
PCBP1 expression in serous ovarian cancer. Expression of PCBP1 (probeset 208620_at) in serous tumors of low malignant potential (LMP) versus malignant serous ovarian tumors [28]. PCBP1 was significantly up-regulated (p = 0.003, Welch's t-test) in malignant tumors. Squares represent the individual samples used in the microarray experiment. Boxplots are overlaid with the lower and upper ends of a box indicating the 25th and 75th percentiles, respectively. The solid black line inside a box indicates the median.

To further investigate the role of PCBP1 in ovarian cancer, we retrieved the transcriptional profiles of an independent ovarian cancer dataset containing 30 tumors of low malignant potential and 60 serous ovarian cancer tumor samples [28] available at Gene Expression Omnibus (http://www.ncbi.nlm.nih.gov/geo/; accession no. GSE12172). Compared to low malignant potential tumors, there was a significant increase in PCBP1 gene expression in malignant ovarian tumors (Figure 6). Furthermore, OncoPrints from the cBioportal for Cancer Genomics (http://www.cbioportal.org) showed that about 10% of all serous malignant ovarian tumors had an altered PCBP1 mRNA expression (data not shown) [29]. The combined observations of elevated protein expression of PCBP1, with the computational analysis highlighting over-representation of PCBP1 protein interactions in this study, as well as the elevated gene expression in a microarray dataset, are suggestive of a role of PCBP1 in ovarian tumor biology, a previously unreported finding.

## Discussion

In order to identify proteins and pathways underlying the differences between benign and malignant ovarian tumors, we performed a comparative proteomic analysis of ovarian tumor tissue and serum, from a limited number of patients using a combination of LC-MS^e^ label-free mass-spectrometry and extensive bioinformatics analyses. Our control group consisted of patients with a benign ovarian tumor instead of healthy women. Therefore, the proteins differentially expressed in our study are more likely to be ovarian tumor-specific instead of reflecting a more general response to disease. We further enhanced robustness of our results by using well matched patient groups and by reducing within-tumor heterogeneity via the use of laser microdissected tumor tissue lysates. In addition we used strict experimental protocols, extensive quality control and state-of-the-art bioinformatics analyses to control for false positive results.

Our study revealed a large number of changes in protein expression in serum and tissue from patients suffering from benign and malignant ovarian tumors. Some proteins were already known to play a role in ovarian cancer, others are new candidates, which may provide new insights into the etiology of the disease or act as potential new disease markers. For a large subset of proteins identified in this study, literature searches confirmed their role in ovarian cancer. For example, Dieplinger et. al. also measured decreased plasma concentrations of Afamin and APOA4 in patients with ovarian cancer, with APOA4 adding independent diagnostic information to CA125 and age for differentiating ovarian cancer from benign and healthy samples [30]. The observed reduction in serum of PON1, that usually mediates enzymatic protection against oxidative stress, may lead to increasing DNA damage and consequently to malignant transformation. It has been suggested before, that SNPs reducing PON1 activity may be associated with an increased risk of epithelial ovarian cancer [31]. In addition, we found a significant decrease of APOA1 in serum of ovarian cancer patients as has been shown before in various studies [32], [33] and in tissue. To our knowledge this significant decrease in tumor tissue samples has not been described yet. The altered serum signature, which is also enriched for other proteins involved in cholesterol and lipid metabolism, suggests that an alteration of these pathways may be beneficial for the cancer cells.

Although substantial efforts have been devoted to detect new serum biomarkers for ovarian cancer, we are the first to describe proteomic analysis in both serum and tissue. This approach may lead to detection of serum proteins directly derived from the tumor, which can give new insights in the pathways active in benign and malignant disease. Only 16 proteins were identified in both serum and tissue, however, with only Apolipoprotein A-I and Serotransferrin being significantly differentially expressed in both. This limited overlap is probably due to the fact that we only detect abundant proteins in both serum and tissue and that abundant tissue proteins have a different concentration in serum and vice versa. Some of the detected proteins are also compartment specific, and therefore only detectable in serum or tissue.

In order to see whether there might be similarities in significantly enriched processes or altered transcription factors within the serum and tissue datasets we performed enrichment analyses using MetaCore. This analysis revealed that 9 out of the top-20 transcription factors associated with key transcriptional regulatory networks in serum were also found in the top-20 of tumor tissue. These results indicate that although the detected proteins only overlap to a limited extent between the 2 datasets, the detected tumor cancer proteome is reflected by the serum proteome at the molecular subnetwork level. This approach may provide another strategy for biomarker discovery.

Amongst others Sp1 and GCR-alpha were detected as potential key transcription factors of the key transcriptional regulatory networks underlying the proteomic signatures in both tissue and serum. The protein encoded by the Sp1 gene is a zinc finger transcription factor that binds to GC-rich motifs of many promoters. It is involved in many cellular processes, including cell differentiation, cell growth, apoptosis, immune response, response to DNA damage, and chromatin remodeling [34]. Post-translational modifications such as phosphorylation, acetylation, glycosylation, and proteolytic processing significantly affect the activity of this protein, which can be an activator or a repressor. Sp1 has been suggested to be responsible for many features of ovarian cancer cells like oncogenic transformation and epithelial to mesenchymal transition for example through activation of KLF8 [35]. Expression of Sp1 is frequently increased in human epithelial ovarian cancers and inhibitors of Sp1-dependent transcription both in vitro and in tumor xenografts have been suggested as interesting candidates for treatment [36].

Receptors for glucocorticoids, like GCR-alpha, are present in tumor cells of almost 90% of ovarian cancer cells and mRNA of GCR-alpha was detected in a wide range of ovarian cancer cell lines [37]. In vitro studies suggests that glucocorticoids may have an adverse effect on outcome in several cancers, including ovarian [38]. However, other investigators have reported favorable effects of GCs in vitro [39]. A study in GCR-positive patients gave no evidence that GCR expression had any prognostic value nor was there any evidence of poorer survival in a small subset of GCR-positive patients who received GC treatment [40]. A recent study has discovered a role for the glucocorticoid receptor within the SLIT glycoprotein ligand and their ROBO receptor pathway. This pathway plays a fundamental role in mammalian development by promoting apoptosis and repulsing aberrant cell migration. SLIT/ROBO expression could be increased by reducing the expression of the glucocorticoid receptor using siRNA. Their findings indicate that in the post-ovulatory phase a role of cortisol may be to temporarily inhibit SLIT/ROBO expression to facilitate regeneration of the ovarian surface epithelium. Therefore this pathway may be a target to develop strategies to manipulate the SLIT/ROBO system in ovarian cancer [41].

Interactome analysis revealed 5 proteins that were significantly overconnected in our tumor tissue signature, meaning they had more connections within the tumor signature than expected by chance and thus are potentially highly relevant in tumor pathophysiology. Two of those, 14-3-3 beta/alpha and 14-3-3 zeta/delta, are members of the 14-3-3 protein family which have been described in previous studies to promote cell survival through suppression of apoptosis [42]. Due to its upregulation in a variety of human tumors and its involvement in cancer progression and treatment resistance, 14-3-3 zeta is currently undergoing extensive investigation as a novel therapeutic target [43].

The other overconnected proteins were actinin-4, HSP60, and PCBP1. Actinin-4 is an isoform of non-muscular-actinin, which enhances cell motility by bundling the actin cytoskeleton [44]. Implications of actinin-4 have been demonstrated in some human malignancies including ovarian cancer. Yamamoto *et al*. have demonstrated high actinin-4 protein expression in 57% of the primary ovarian carcinomas [45], [46]. High expression was associated with serous histology, high histological grade, and poor patient outcome.

Heat shock proteins (HSPs), like HSP60, are important players in protein homeostasis and cell and tissue physiology, as well as in protection against stressors [47]. HSPs intervene not only in protein folding, refolding, trafficking and degradation but also in the regulation of cell growth and differentiation, apoptosis and cell-to-cell crosstalk, inflammation, and tissue repair [47], [48]. The importance of HSPs has come into focus in the past few years because it has been realized that they can be pathogenic factors in a variety of conditions. Among these pathologies there are various forms of cancer, in which the proteins are normal but work in favor of the tumor rather than protect the patient [49]. In these conditions, HSPs enhance tumor cell survival and growth by inhibiting apoptosis and the anti-tumor immune response, or by promoting neoangiogenesis [50]. In our study, several of the HSPs had altered expression. Besides the overconnected HSP60, six HSP family members were found to be upregulated in the malignant tissue samples, further highlighting their importance in ovarian cancer biology.

**Figure 7:**
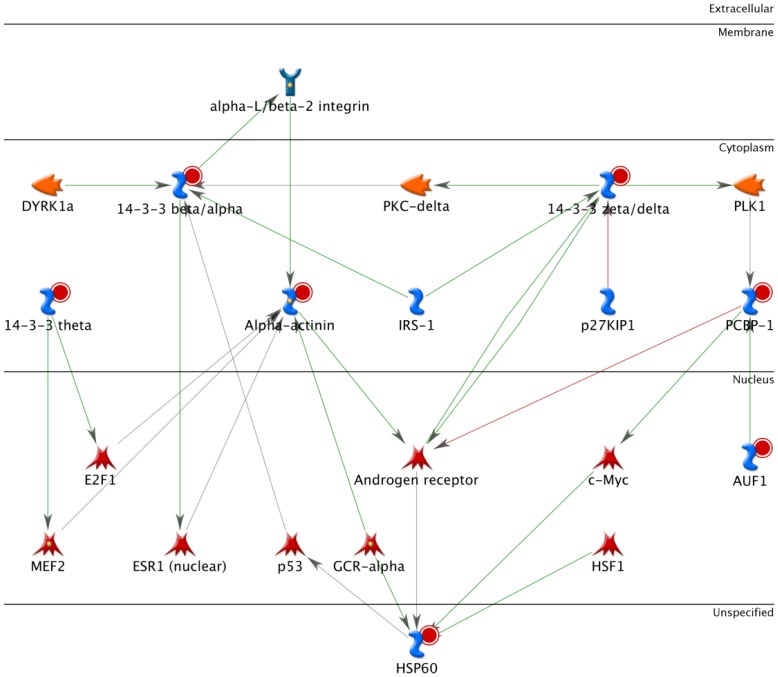
Interactions between overconnected proteins in tumor tissue. A network built in MetaCore using the 5 overconnected proteins, PCBP-1, Alpha-actinin 4, HSP60, 14-3-3 beta/alpha and 14-3-3 zeta/delta as seed nodes yielded a highly interconnected network amongst the seed nodes and implicated a role for androgen receptor signaling.

The fifth overconnected protein, PCBP1, was originally discovered as an RNA-binding protein, which participates in mRNA processing at multiple steps. Subsequent studies showed that PCBP1 possesses multiple functions in transcription, splicing, and translation. The protein is capable of switching among its various functions depending upon its state of phosphorylation as well as its cellular localization, and can therefore act as a corepressor and a coactivator in response to different environmental signals [51]. In the cBioPortal database, almost all neighboring genes connected to PCBP1 have altered expression in more than 20% of the serous ovarian cancer samples (data not shown) providing further evidence of a critical role for PCBP1 in the pathophysiological processes underlying malignant ovarian tumors. Importantly, we further implicated PCBP1 within ovarian tumor biology by the fact that the network built in MetaCore using the 5 overconnected proteins as seed notes and their nearest neighbors, generated a network highly interconnecting all 5 seed nodes. The results of this network analysis also revealed an interesting connection to the androgen receptor-signaling pathway (Figure 7). As PCBP1 has been previously found to regulate the androgen receptor in androgen-responsive cells, like the prostate cancer LNCaP cells [52], this connection might be underlying the function of PCBP1 in ovarian tumor biology as well. Overall this network analysis, which attempts to reconstruct the biological mechanism underlying the proteomics profile, provides further mechanistic insights for future validation as well as potential targets of intervention.

In summary, by quantifying protein expression in serum and tissue we found proteins differentially expressed between benign and malignant samples. These included proteins previously identified to play a role in ovarian cancer, but also candidates that had not been implicated in the disease process before. We observed not only a significant decrease in serum APOAI and Serotransferrin, as previously reported, but provide evidence for decreased expression in the tumor site as well. Although the serum and tissue signatures had only 16 proteins in common, we highlighted common transcription factors potentially relevant for changes in both the serum and tumor proteome through in-silico analyses. Important roles are suggested for GCR-alpha and Sp1. In addition, our results implicate an as yet unknown role for PCBP1 in ovarian cancer. We hypothesize that the protein network including PCBP1 contributes to the malignant properties of ovarian tumors, possibly through regulation of the androgen receptor. Further experiments are warranted to unravel the precise mechanism of PCBP1 in ovarian cancer.

## Supporting Information

Table S1 **List of proteins identified in serum and tissue of ovarian cancer vs. benign patients.** List of file names as reported in ProteomeXchange with the corresponding sample annotation in worksheet named ‘Filenames’. List of all the measured samples with a complete list of protein identifications in worksheet named ‘Raw data’ together with separate worksheets containing the filtered data named ‘Serum (filtered)’ and ‘Tissue (filtered)’. Detailed information on the annotation of the proteins and the outcome of the differential expression analysis is provided.
(XLS)

Table S2 **Protein set enrichment analysis using the Cytoscape plugin “ClueGO”.** The proteins associated with significant GO categories are displayed as well as the p-value associated with the GO term. The first four categories are mostly specific to the serum proteome signature while the other categories represent biological processes that are mainly specific to the tissue proteome signature.
(XLS)

Table S3 **Serum and tissue transcriptional regulation network list.** The column ‘GO processes’ states the different biological processes associated with the network. The total number of objects in the network is indicated in the ‘Total nodes’ column. The p-values represent the probability of intersection between the experimental signature and the prebuilt content in MetaCore. The column ‘zScore’ gives the level of saturation of the networks taking into account the size of the database, the number of objects in the subnetwork and the number of objects in the signature used to construct the network. The higher the z-score the more saturated a subnetwork is. The transcriptional regulation networks are ranked according to p-value.
(XLS)

Text S1 **Batch effect and batch correction for the tissue samples.**
(DOC)
