# Supplementary material for: Label-Free LC-MSe in Tissue and Serum Reveals Protein Networks Underlying Differences between Benign and Malignant Serous Ovarian Tumors
Source: PLoS One. 2014 Sep 29;9(9):e108046. doi: 10.1371/journal.pone.0108046 (PMC4180266; doi:10.1371/journal.pone.0108046)
Supplement: Text S1 — Batch effect and batch correction for the tissue samples. (DOC) [file pone.0108046.s004.doc]

**Supplementary Information: Label-free LC-MS^e^ in tissue and serum reveals protein networks underlying differences between benign and malignant serous ovarian tumors**

Wouter Wegdam, Carmen A. Argmann, Gertjan Kramer, Johannes P. Vissers, Marrije R. Buist, Gemma G. Kenter, Johannes M.F.G. Aerts, Danielle Meijer, Perry D. Moerland

Data quality control (QC) is an important step when performing any proteomic study. A combination of metrics and techniques for assessing data quality can help to identify systematic technical effects and outlier samples. We performed extensive QC on the using the arrayQualityMetrics R/Bioconductor package [1]. This package has been developed within a European project for improving standards and standardisation of microarray technology and data analysis (EMERALD), but is also useful for QC of proteomic data. The quality metrics included in the package assess reproducibility, identify apparent outlier arrays, and compute measures of signal-to-noise ratio. Performing QC using arrayQualityMetrics on the log-transformed normalized serum data indicated no outlying samples or other technical issues. The tissue samples were measured in two separate batches (Supporting information Table S1). QC showed that although the major distinction is between benign and malignant samples, within these groups a clear separation due to batch effect is present (Supplementary Figure S1A). Here, the batch effect might lead to

**A**

**
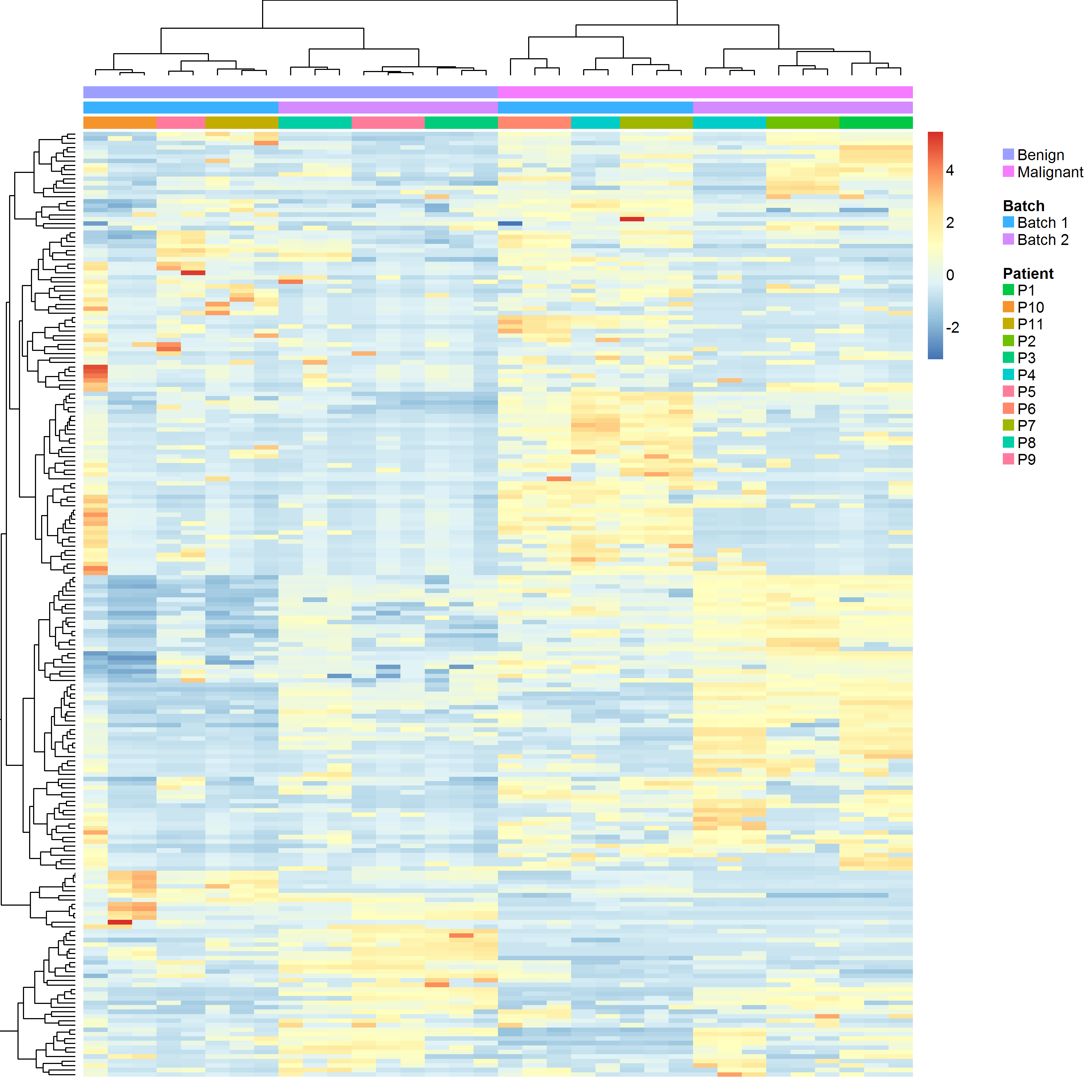
**

**B**

**
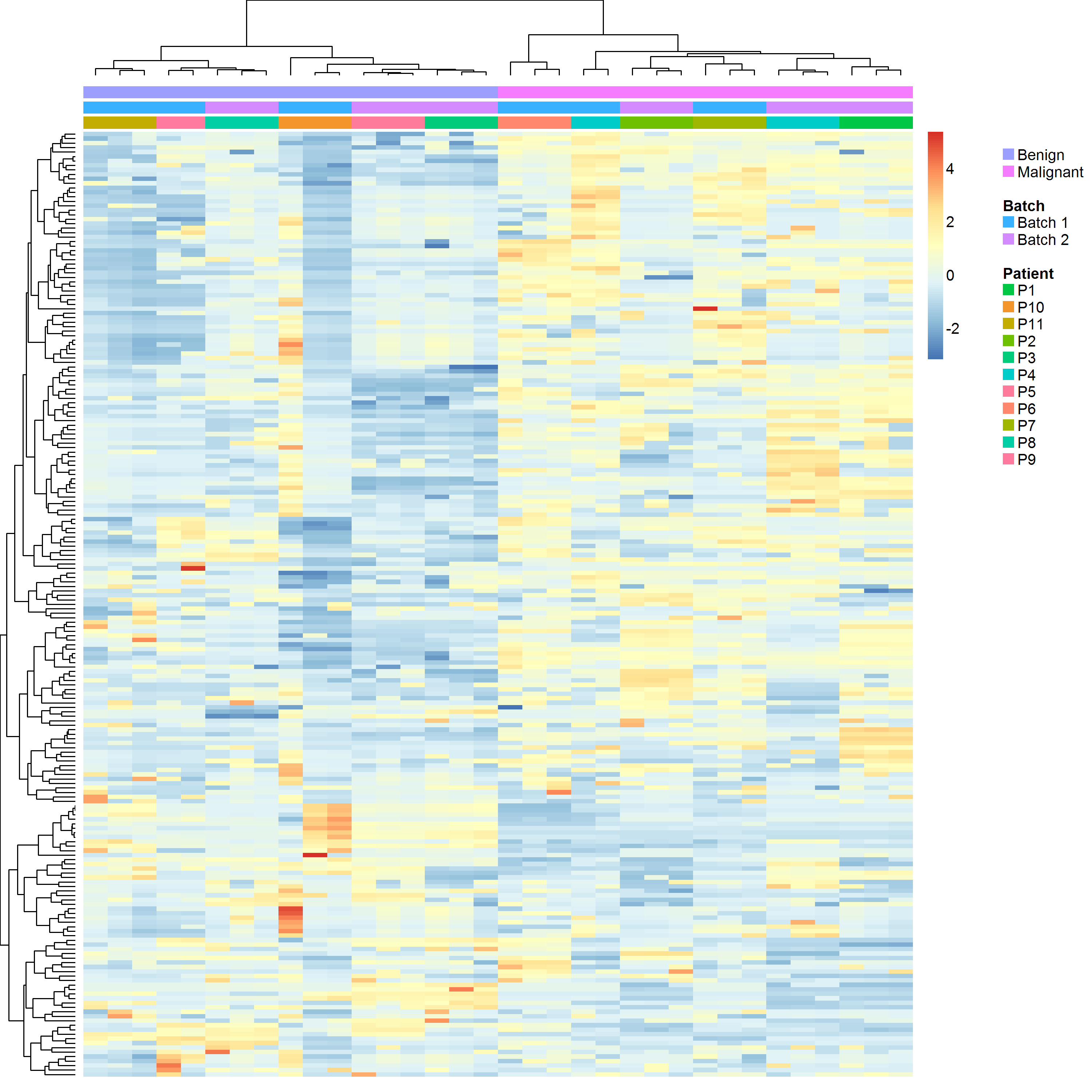
**

**Figure S1.** Unsupervised hierarchical clustering (complete linkage, Pearson correlation distance) was performed on the profiles of the 209 proteins detected in tumor tissue samples. The colour bar indicates standardized protein expression levels (dark orange indicates relatively higher expression; dark blue indicates relatively lower expression). **A**) Heatmap of normalized tissue data. **B**) Heatmap of batch corrected normalized tissue data. Note that multiple replicates were measured for each patient.

larger within-group variance and therefore underestimation of the number of differentially expressed proteins. We corrected for the batch effect in two different ways. First, for the determination of differentially expressed proteins a linear model was fit on the normalized data for each identified protein. The linear model contained the two conditions (benign and malignant) as explanatory variable and a batch factor as covariate. This way the comparison malignant vs. benign was adjusted for differences between the batches. Second, prior to unsupervised analyses such as clustering the same linear model was used to explicitly remove the component due to the batch effects (function ‘removeBatchEffect’, R/Bioconductor package limma). The resulting data indeed did not show a batch effect, while the clear distinction between benign and malignant was conserved (Supplementary Figure S1B). Note also the good reproducibility of the technical replicate samples for each patient.

The reader is referred to the Experimental Procedures section in the main text, for a detailed description of other aspects of the statistical and bioinformatics analyses.

**References**

[1] Kauffmann A, Gentleman R, Huber W. arrayQualityMetrics--a Bioconductor package for quality assessment of microarray data. Bioinformatics. 2009;25(3):415-6.
